# Social Media Use, Psychological Distress and Knowledge, Attitude, and Practices Regarding the COVID-19 Among a Sample of the Population of Pakistan

**DOI:** 10.3389/fmed.2021.754121

**Published:** 2021-10-20

**Authors:** Muhammad Rizwan, Tauseef Ahmad, Xuehong Qi, Manal Abdulaziz Murad, Mukhtiar Baig, Abdullah Khalid Sagga, Saba Tariq, Fizzah Baig, Rozina Naz, Jin Hui

**Affiliations:** ^1^School of Education Sciences, Nanjing Normal University, Nanjing, China; ^2^Department of Epidemiology and Health Statistics, School of Public Health, Southeast University, Nanjing, China; ^3^Key Laboratory of Environmental Medicine Engineering, Ministry of Education, School of Public Health, Southeast University, Nanjing, China; ^4^Department of Family Medicine, Faculty of Medicine, King Abdulaziz University, Jeddah, Saudi Arabia; ^5^Department of Clinical Biochemistry, Faculty of Medicine Rabigh, King Abdulaziz University, Jeddah, Saudi Arabia; ^6^General Dentist, Assistant Professor for Primary Health Care, Medical Program for Chronic Disease, General Department, Ministry of Health, Riyadh, Saudi Arabia; ^7^Department of Pharmacology, University Medical & Dental College, The University of Faisalabad, Faisalabad, Pakistan; ^8^Ziauddin Medical College, Ziauddin University, Karachi, Pakistan; ^9^Department of Psychology, Institute of Southern Punjab, Multan, Pakistan

**Keywords:** knowledge, practices, attitude, COVID-19, depression, anxiety, stress, social media

## Abstract

This study was conducted to assess social media (SM) use, psychological distress, and knowledge, attitude, and practices (KAP) regarding the coronavirus disease 2019 (COVID-19) among a sample of the population of Pakistan. A cross-sectional study was conducted in Multan, Pakistan between April and May 2020. Demographics details, SM use, psychological distress, and KAP on the COVID-19 were investigated. A total of 800 respondents were analyzed out of which 33.5% (*n* = 268) were women. No gender-wise difference was found in the terms of SM use and duration of SM use. Women were prone to have psychological disorders such as anxiety, depression, and stress than men in the current COVID-19 pandemic. The mean knowledge score of men was significantly higher compared to women (18.69 ± 4.20 vs. 16.89 ± 3.04, *p* < 0.001), while the mean score of the attitude and practices on the COVID-19 prevention measures was significantly better in women (*p* = 0.012 and *p* < 0.001, respectively). The psychological problems such as anxiety (*p* < 0.001) and depression scores (*p* = 0.033) were higher among women than men. The stress score was also higher in women but not significantly higher (*p* = 0.079). The knowledge was significantly correlated with attitude, anxiety, depression, and stress. The regression analysis showed that the COVID-19-related KAPs are the predictors of psychological suffering of an individual. The female gender was positively associated with anxiety and depression. The SM use was the predictor of the stress. Male respondents had significantly more knowledge of the COVID-19 than female respondents, but women had significantly better attitudes and practiced the COVID-19 prevention measures. Gender is a significant determinant of psychological distress and KAP about the COVID-19. The government has already taken significant steps to limit the spread of the disease; however, much more effort is required to tackle this COVID-19 pandemic.

## Introduction

Coronavirus disease 2019 (COVID-19) is an unprecedented global health emergency that was first reported in Wuhan, China in December 2019 and spread globally like a wave (1). Since the onset of the COVID-19 pandemic, the number of infections and deaths has been escalating at an alarming and uncontrollable rate across the world (2); after mutations, it is also causing second and third waves globally. As on September 10, 2021 (2:30 a.m., Beijing, China time), a total of 222,862,499 cases and 4,600,959 deaths have been reported due to the COVID-19 worldwide (https://coronavirus.jhu.edu/map.html). Several vaccine candidates have been available, but people have doubts and hesitancy to get vaccinated (3).

Numerous benefits and global use of social media (SM) have made it part and parcel of lives of the peoples. The impact of SM in molding perceptions of the people and disseminating information and disinformation about the COVID-19 epidemic is noteworthy. WhatsApp (WA), Facebook (FB), Twitter (TW), YouTube, and Instagram (Insg) are the most popularly and widely used SM platforms worldwide (4). SM is a strong tool and a source of timely information about significant problems and health hazards, but it is not always reliable. The information provided by many SM platforms is untrustworthy and it is impossible to differentiate between the rumors and facts (5). The mental health sufferings due to the COVID-19 are widely observed (4, 6, 7). Panic, worry, insecurity, and stress have erupted as a result of the widespread lockdown. People in Pakistan have been quarantined at home because of the COVID-19 and self-isolation has put them under physical and psychological strain. In addition, many people have lost their employment and are living in terrible circumstances. The effects of the pandemic in Pakistan have frightened the masses and have increased the workload of medical staff (8, 9).

Coronavirus disease 2019 pandemic has created a lot of chaos and tilted most of the economies of the countries worldwide (1). Moreover, the fear of the COVID-19 is essentially leading to suicide observed in India (10) and Pakistan (11). It is expected that the mental health crisis in Pakistan will become a severe problem that persists even after the COVID-19. As a result, it is critical to adopt effective efforts to address its mental health issues. Mental challenges and stress have been linked to prevalent mental disorders such as anxiety and depression in the different regions of the world (12). The record of Pakistan in mental health is not notable, while over 50 million people are estimated to be affected by mental health disorders. They are taken care of by only 500 psychiatrists with a patient-to-psychiatrist ratio of 1 to 100,000 (13).

To control such COVID-19 pandemic, precise and timely monitoring of factors involved in speeding the transmission with vaccination are of great importance. A factual analysis of the different facts such as racial, cultural, geographical, and religious variables could assist in developing much better and effective preventive measures in controlling the spread of this disease. It has been reported that understanding the public perception about knowledge, attitude, and practices (KAP) to infectious disease could help the health authorities and policymakers to identify the gaps and solutions to fill that gap. It also helps the affected country to make the proper rules to fight against such pandemic (7, 14).

To combat the COVID-19, the government of Pakistan has dedicated the resources to fight effectively against this outbreak and prevent and promote health through public awareness. Apart from this, the Pakistan government has taken some unprecedented measures to control the COVID-19 transmission by implementing lockdown that includes suspension of public transportation, closing all the educational institutes, closing all the shopping markets, and isolation and care for the COVID-19 suspected and infected peoples (14, 15).

Controlling and containing the current COVID-19 pandemic depends mainly on the adherence of the people to follow the instructions over a long period. However, according to the KAP theory, adherence commitment of the people can be affected by their KAP. Therefore, there is a great need to understand the public approach toward the COVID-19 in this critical situation to facilitate outbreak management. This study was designed to assess SM use, psychological distress, and the KAP regarding the COVID-19 among a sample of the population of Pakistan. The results of this study may assist the health authorities in making effective and robust strategies and recommendations and getting the community involved in controlling the COVID-19.

## Methods

### Study Design

A cross-sectional descriptive study was conducted over the general population from Multan, Pakistan from April 11 to May 30, 2020.

### Data Tools and Collection

Due to lockdown, a community-based sampling survey was not feasible; therefore, it was decided to collect it online. Due to the lack of internet facilities in some rural areas, researchers personally approached the communities relying on networks of the authors by using a convenient sampling technique. Participants more than 20 years of age with no learning disabilities and willing to participate were given a questionnaire to fill independently. All the tools were translated into the native language, Urdu, and the reliability of the scales showed that the questionnaire is culturally fit for the indigenous population.

Institutional and national ethical guidelines as well as the Helsinki Declaration were followed throughout the data collection process. It was ensured that the data was kept anonymous and undisclosed. Creative Research Systems Survey Software Sample Size Calculator calculated a representative sample size of 665 at a confidence level of 99%, a margin of error of 5%, and population size of 1.9 million to achieve the required sample size and statistical power size. In order to increase the validity and generalizability of the study, the estimated sample size was inflated to reach as many of the participants as possible. The consent statement was included at the beginning of the questionnaire and the information was garnered voluntarily. We assured all the participants that this information would be used solely for research purposes and that their identities would not be revealed. The investigators took verbal informed consent from all the study participants.

### Questionnaire

The questionnaire was comprised of three sections: demographic details and use of SM, KAP, and psychological distress. The demographic variables included age, sex, marital status, educational attainment, occupation, monthly income, family structure, and place of current residence. The questionnaire was adopted from Zhong et al. (16) with slight modification. The questionnaire consisted of 12 questions of the knowledge domain; among them, four questions were related to the clinical presentations of the COVID-19 (K1-K4), three questions were related to the mode of transmission (K5-K7), and five questions were related to the control and prevention of the disease (K8-K12). The alternative responses of all these questions were “True,” “False,” or “I don't know.” The true response was assigned two points, the false response was assigned one point, and the “don't know” response was assigned a zero point. The total knowledge score ranged between 0 and 24 with a high score indicates better knowledge about the COVID-19.

The attitude was assessed by the two questions (A1-A2) about the final control and confidence in winning the battle against the COVID-19. The questions within the attitude domain were scored in the same way as in the knowledge domain (i.e., ranged between 0 and 4). The practice domain consisted of two questions assessing behaviors of respondents about social distancing and the use of the mask. The response alternatives were “Yes” or “No” and a score of one was given to the response that adheres to health protocol, otherwise zero. In this study, Cronbach's alpha coefficient indicated an acceptable internal consistency of the knowledge questionnaire (Cronbach's alpha = 0.71).

Two questions (A1-A2) about the agreement on final control of the COVID-19 and confidence in winning the battle against COVID-19 were used to gauge attitudes of the people toward the COVID-19 pandemic. SM and its frequency were measured through the items of KAP that is used to examine the trend of using SM among the people during the COVID-19.

The following questions gathered the information related to SM as follows: “Do you use SM” (Yes/No); “Which SM do you use?” (1 = FB, 2 = WA, and 3 = Insg); “How much time do you spend on SM every day?” (1 = “1 hour,” 2 = “3 hours,” 3 = “5 hours,” and 4 = “8 hours”); “How would you describe your frequency of SM use?” (1 = “too much,” 2 = “some extent,” 3 = “very little,” and 4 = “not at all”); “Do you believe in SM information? (1 = “too much,” 2 = “some extent,” 3 = “very little,” and 4 = “not at all”), and also other questions.

Anxiety, depression, and stress were measured collectively through the Psychological Distress Scale for Adults (PDS-A) (17) that is recently developed in culture of Pakistan. It is comprised of three dimensions: anxiety (cognitive, physical, and behavioral); depression (cognitive, physical, and behavioral); and stress (cognitive, physical, and behavioral). Total items were 38 with a four-point response rate. It is a validated questionnaire in Pakistan and its alpha reliability is 0.92.

### Statistical Analysis

The descriptive statistics were used for measuring the frequencies and percentages of the correct answers of the KAP domain. Duration of SM use/day, scores of the KAP, and psychological problems (anxiety, depression, and stress) were compared between the genders by using a *t*-test, while the use of SM between genders was compared by chi-squared test. The Pearson correlation coefficient was calculated among the different variables and multiple linear regression analysis was employed to explore the anxiety, depression, and stress predictors. All the data analysis were applied through the SPSS-21 version for windows. The significance was judged notably if the *p*-value was less than 0.05.

## Results

### Characteristics of the Participants

The response rate of this study was 67% (800/1,200). Among them, 66.5% (*n* = 532) were men. Most of the participants (90.8%, *n* = 726) were using SM and the most common SM used by the participants was WA followed by the FB and Insg. The prevalence of the anxiety, stress, and depression was 66.5% (*n* = 532), 60.5% (*n* = 484), and 59.7% (*n* = 478), respectively. Other general characteristics of the study participants are shown in Table 1.

**Table 1:** Demographic characteristics of the study participants (*n* = 800).

**Characteristics**	**Group**	***n* (%)**
Gender	Male	532 (66.5)
	Female	268 (33.5)
Age, years	20–30	441 (55.1)
	31–40	249 (31.2)
	41–50	66 (8.3)
	51–60	37 (4.6)
	>60	7 (0.9)
Family structure	Joint	512 (64)
	Nuclear	288 (36)
Monthly income, Rupees	15,000	96 (12)
	16,000–30,000	260 (32.5)
	31,000–45,000	132 (16.5)
	46,000–60,000	138 (17.2)
	Above 60,000	174 (21.8)
Use of social media	Yes	726 (90.8)
	No	74 (9.3)
Types of social media used	FB	128 (22.8)
	WA	592 (74)
	Insg	26 (3.2)
Psychological distress	Anxiety	532 (66.5)
	Stress	484 (60.5)
	Depression	478 (59.8)
Marital Status	Married	615 (76.8)
	Unmarried	175 (21.8)
	Widow	10 (2.4)
Occupational	Employed	278 (34.7)
	Unemployed	67 (8.3)
	Business	298 (37.2)
	Farming	157 (19.6)
Education	Yes	567 (70.8)
	No	233 (29.1)

*WA, WhatsApp; FB, Facebook; TW, Twitter; Insg, Instagram*.

### Duration of SM Use

**Figure 1:**
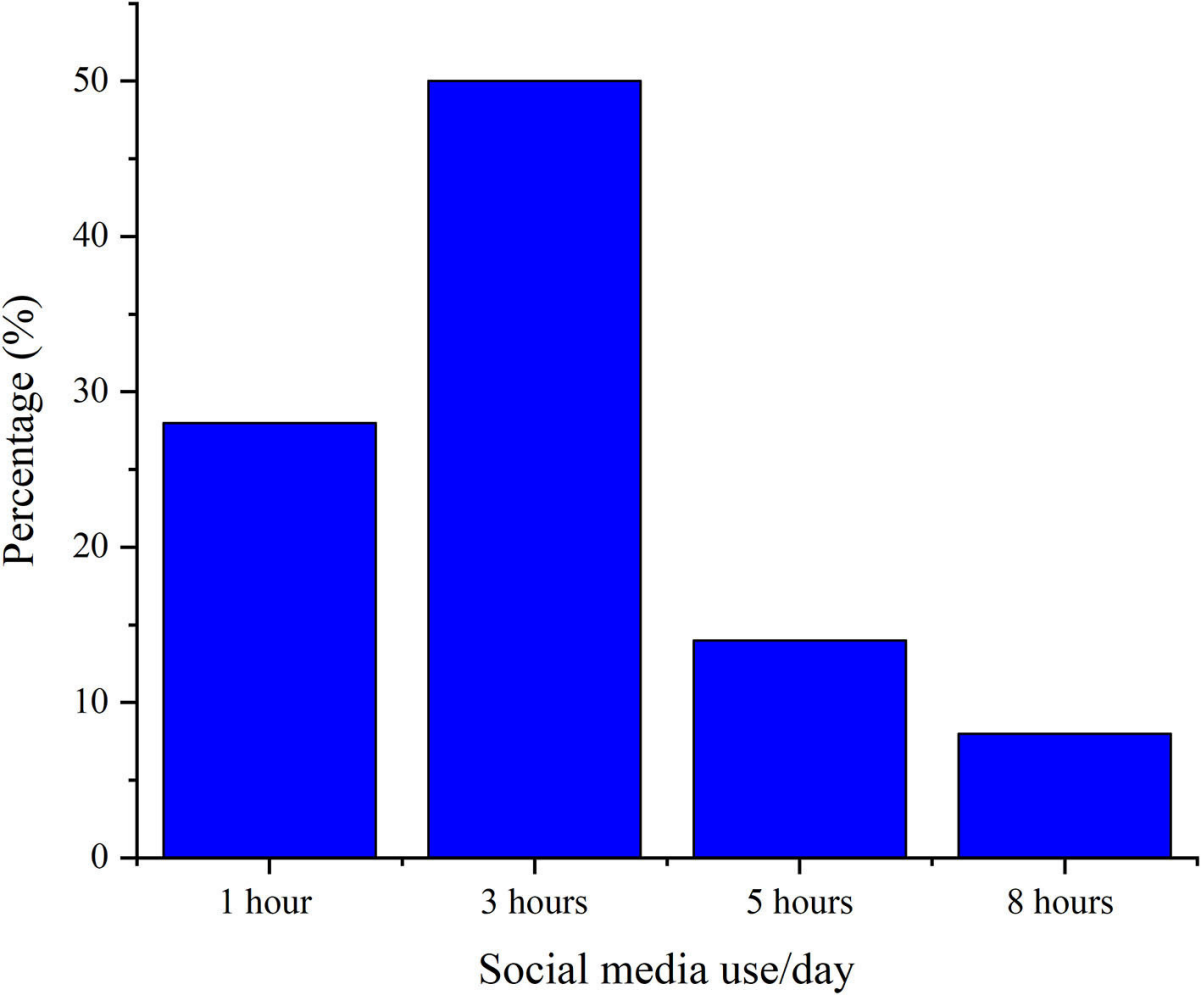
Social media use per day among the study participants (*n* = 726).

Among the study participants, the use of SM per day was one hour in 28.2% (*n* = 205), three hours in 50% (*n* = 363), five hours in 14.2% (*n* = 103), and eight hours in 7.5% (*n* = 55) (Figure 1).

### Gender and SM Use

**Figure 2:**
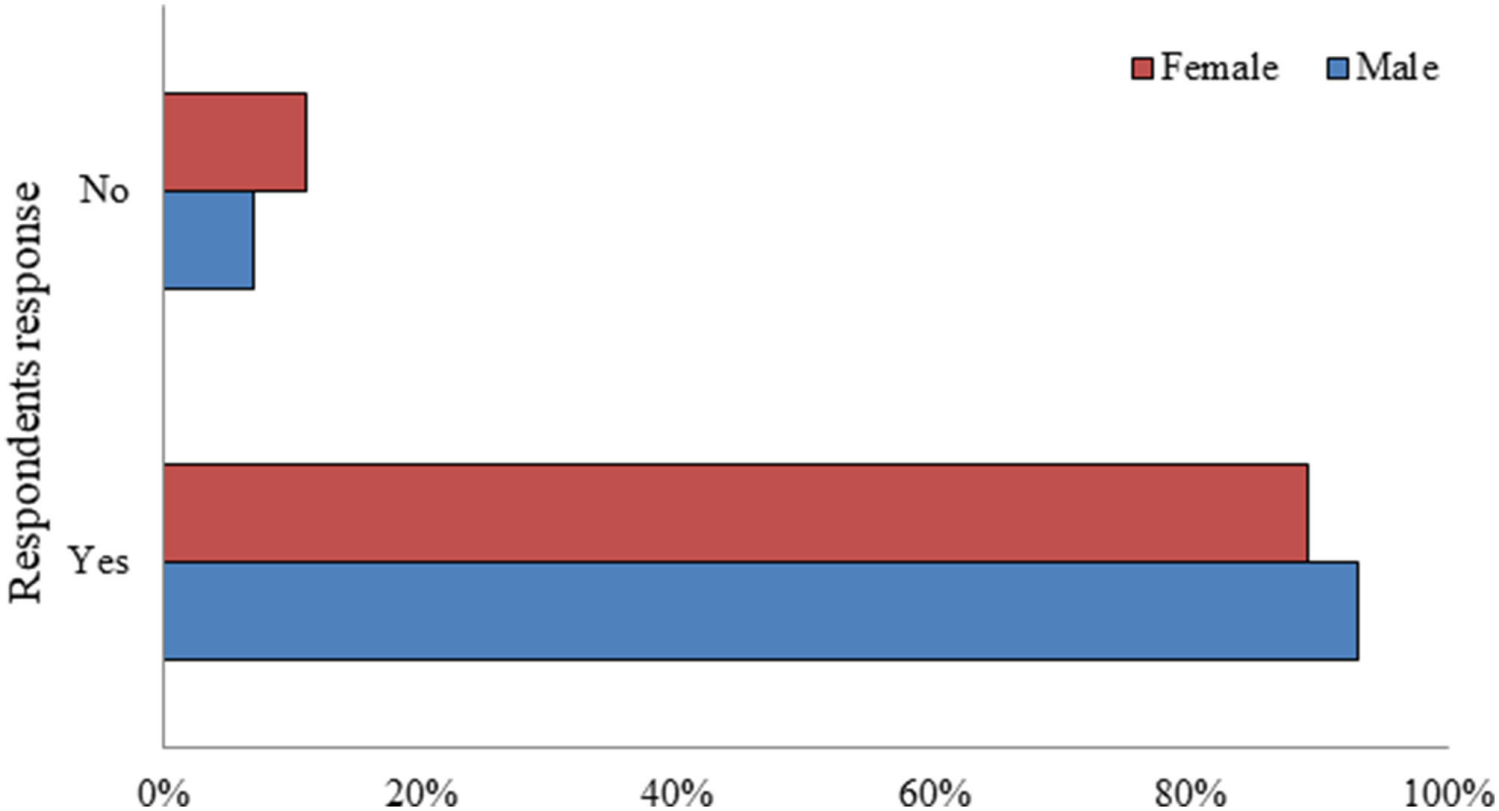
Gender-wise comparison of social media use among the study participants (*n* = 726).

Gender-wise comparison of SM use among the study participants showed no significant difference between the male and female subjects (*p* = 0.08) (Figure 2).

### Comparison of Duration of Social Media Use, Knowledge, Attitude, and Practices Regarding the COVID-19, and the Anxiety, Depression, and Stress Levels Between the Genders

No significant difference was observed in the duration of SM use per day between the men and women. The mean score of the COVID-19-related knowledge was significantly better among the male respondents compared to women respondents (score 18.69 ± 4.20 vs. 16.89 ± 3.04, *p* < 0.001). The mean score of the attitude and practices on the COVID-19 prevention measures was significantly better in women (*p* = 0.012 and *p* < 0.001, respectively). The psychological problems such as anxiety, stress, and depression scores were higher among the women than men (Table 2).

**Table 2:** Comparison of duration of social media use, knowledge, attitude, and practices regarding the coronavirus disease 2019 (COVID-19), and anxiety, depression, and stress levels between the genders (*n* = 800).

**Variable**	**Gender**	***n***	**Mean score**	**Std. Deviation**	***t*-test**	***p*-value**
Duration of SM use	Male	490	3.19	1.72		0.059
	Female	236	2.90	2.17		
Knowledge	Male	532	18.69	4.20	6.214	<0.001
	Female	268	16.89	3.04		
Attitude	Male	532	2.33	0.68	−2.521	0.012
	Female	268	2.47	0.83		
Practice	Male	532	2.75	0.55	−5.723	<0.001
	Female	268	2.98	0.47		
Anxiety	Male	532	31.91	7.78	−3.695	<0.001
	Female	268	33.98	6.80		
Stress	Male	532	23.54	7.62	−2.140	0.033
	Female	268	24.68	6.02		
Depression	Male	532	25.90	8.35	−1.760	0.079
	Female	268	26.97	7.37		

### Correlation of the Study Variables

A positive relationship of knowledge was found with attitude, anxiety, depression, and stress. Unexpectedly, knowledge of the COVID-19 was negatively correlated with practice. A significant positive correlation between anxiety, depression, and stress was observed (Table 3).

**Table 3:** Correlation between the gender, age, family structure, social media use and duration, knowledge, attitude, and practices regarding the coronavirus disease 2019 (COVID-19), and anxiety, depression, and stress levels (*n* = 800).

**Variable**	**1**	**2**	**3**	**4**	**5**	**6**
1. Knowledge	1					
2. Attitude	0.132^**^	1				
3. Practice	−0.202^**^	−0.026	1			
4. Anxiety	0.217^**^	−0.142^**^	0.085^*^	1		
5. Depression	0.380^**^	−0.043	−0.044	0.652^**^	1	
6. Stress	0.444^**^	0.015	0.043	0.721^**^	0.767^**^	1

* *p-value is significant at <0.05*,

** *p-value is significant at <0.01*.

### Predictors of Anxiety, Depression, and Stress

Table 4 demonstrates that the COVID-19-related KAP has a substantial influence on psychological suffering of an individual. The female gender was associated with anxiety and depression. The SM use was the predictor of the stress.

**Table 4:** Predictors of anxiety, depression, and stress (multiple linear regression analysis).

**Variables**	**Anxiety**			**Depression**			**Stress**		
	**B**	**Std. Error**	***P*-value**	**B**	**Std. Error**	***P*-value**	**B**	**Std. Error**	***P*-value**
Gender	−1.502	0.403	0.000^*^	−1.118	0.362	0.002^*^	0.672	0.359	0.061
Family structure	0.740	0.387	0.000^*^	−0.850	0.346	0.014^*^	0.212	0.342	0.536
SM use	−0.820	0.677	0.056	−0.634	0.606	0.296	−2.164	0.593	0.000^*^
Duration	0.331	0.098	0.226	0.139	0.089	0.116	−0.086	0.087	0.326
Knowledge	−0.151	0.051	0.001^*^	0.143	0.046	0.002^*^	0.350	0.044	0.000^*^
Attitude	−1.333	0.240	0.003^*^	−0.449	0.218	0.040^*^	0.631	0.215	0.003^*^
Practice	0.236	0.344	0.000^*^	−1.226	0.305	0.000^*^	0.805	0.302	0.008^*^
Anxiety				0.186	0.031	0.000^*^	0.419	0.028	0.000^*^
Depression	0.232	0.039	0.000^*^				0.498	0.030	0.000^*^
Stress	0.538	0.035	0.000^*^	0.512	0.031	0.000^*^			
Adjusted R^2^	0.583			0.627			0.718		

* *p <0.05 is significant*.

## Discussion

Our results showed no significant difference in SM use and duration of SM use per day between the men and women. Women had higher psychological issues such as anxiety, stress, and depression compared to men. The prevalence of anxiety, stress, and depression was 66.5, 60.5, and 59.7%, respectively, among our study participants.

Similar to the present study, a Chinese study reported that 94 and 96% of men and women, respectively, were using SM for obtaining the COVID-19 information (no significant difference) (18). They also reported higher levels of psychological issues such as anxiety, stress, and depression among the women than men. A recent Pakistani survey revealed that SM was the principal source of the COVID-19 information among the study participants and more than half of the studied population had insufficient knowledge about this situation (4). They further suggested that those who believed in SM had more misunderstandings. It indicates that the misinformation propagated through these sources influenced perceptions and knowledge of the peoples (4). An Iraqi study found a substantial positive association between self-reported SM use and the spread of COVID-19 fear (19). According to a Chinese study, the frequency of SM usage was a good predictor of COVID-19 prevention activities of an individual (20). In contrast to our results, an Indian study reported a much lower prevalence of anxiety (11.6%), stress (28%), and depression (25%) among the study participants during the COVID-19 (21). They also observed that the male gender was more likely to be anxious (21). According to a Turkish study, women were more mentally afflicted by the COVID-19 pandemic (22). Another study reported that after the pandemics, women had been acknowledged as a major predictor for the symptoms of posttraumatic stress disorder (23).

A Chinese study reported that approximately one-third of the adults and health professionals spend ≥ 2 h per day on the COVID-19 news on SM. People having close contact in the patients with the COVID-19 and spending ≥ 2 h per day on the COVID-19 news via SM were linked to the probable anxiety and depression (24). They also reported that social support was related to a lower likelihood of anxiety and depression in adults. It shows that people in the community need social support and resolution of their grievances to overcome their COVID-19-related depression, anxiety, and stress. Less use of SM could be helpful. The amount of time spent on SM news connected to the COVID-19 was substantially correlated with the occurrence of these mental health disorders, which is likely to be caused by excessive media attention (25). A recent Pakistani study reported that 90% of the study participants used SM to get the COVID-19 information. The majority of them had various types of misconceptions (4). They further reported that this pandemic had affected most of the mental and psychological well-being of the participants. One of the key stressors of anxiety and stress was the financial strain imposed by extensive quarantine (25).

If appropriate, SM utilization should be integrated into global pandemic planning and response while minimizing the unfavorable impact (24). Gao et al. showed a high frequency of the mental health disorders associated with SM exposure during the COVID-19 pandemic (26). Individuals with mental symptoms also experience problems in getting required medical aid for a variety of reasons including the conversion of some hospitals into the pandemic hospitals and the inability of the psychiatric clinics to provide active treatment or the reduction in the number of patients being examined for the security reasons and the risk of the virus-related hospital environments (22).

This study revealed a positive relationship of knowledge with attitude, anxiety, depression, and stress. A significant positive correlation between anxiety, depression, and stress was also observed.

The regression analysis demonstrated that COVID-19-related KAPs are the predictors of psychological suffering of an individual. The female gender was positively associated with anxiety and depression. The SM use was the predictor of the stress. In contrast to our study, a study observed that no significant link exists between knowledge and depression, anxiety, and stress (27). They also reported that girls have higher levels of depression and stress than boys. However, in this study, all the participants were students, while our participants were general public; therefore, it could be one of the reasons for the difference in the results. In contrast to our results, a Turkish study reported no association between knowledge and mental health (28). In an Indian study, more than 80% of the individuals reported a perceived need for mental healthcare (29). The Turkish study findings imply that those with low perceived severity, strong self-efficacy, and participation in the COVID-19 prevention practices have improved mental health during times of crisis (28).

Our results suggest that men had better knowledge, while women had better attitudes and practicing behavior against the COVID-19. Similar results have also been reported in Iran, Pakistan, and Saudi Arabia (7, 15, 30). The reason behind this high knowledge among the men compared to women could be attributed to: (a) more easy access to print electronic, and SM updates about the severity of the pandemic by the men compared with women; (b) men have more habits of reading newspapers or listening news compared with women and in accordance with the reported literature (31); (c) tea shops in Pakistan are also considered a great place for the men to spend a lot of time in discussing various social issues, and (d) most of the men visit the mosques five times a day to offer their prayers and find time to share social news as well.

Therefore, there is a great need to improve more health education practices in the population, particularly among the women. The optimistic attitude of the public regarding COVID-19 pandemic spread could be considered by following the state guidelines or control measures such as traffic limits, the shutdown of cities, social distancing, avoiding unnecessary traveling, saving health, and other essential service resources. All these measures could only be enhanced by knowledge, attitudes, and confidence of the people. Most of the respondents had an optimistic attitude toward the COVID-19 pandemic, as 78.8% of the respondents believed that this COVID-19 pandemic would be controlled and 95.2% of the respondents believe that Pakistan would win the battle against the COVID-19. Similar positive attitudes were found in the previous reports from Saudi Arabia and Pakistan (7, 14, 32).

There were several KAP gaps among the study participants; however, immunization is the most effective way to fight against the COVID-19 pandemic (33–35) and it is social responsibility of every individual to get vaccinated.

## Recommendations and Future Implications

However, the Pakistani community appreciates too little about infection, especially when viewed against this potential global threat scale. Therefore, after relaxing in lockdown, the rate of transmission may increase faster during the next wave. This would further aggravate the low vaccination rate in the country because of several reasons. The main hurdles in controlling the transmission of the COVID-19 and getting vaccinated include poor health literacy and the social and cultural mindsets of a major proportion of the population. This public attitude is alarming and the authorities and public health agencies must respond promptly and aggressively on such issues to control the current COVID-19 pandemic and get ready for further outbreaks.

It is argued that too much awareness and the disease regarding information in the community create panic; but, in our view, it overweighs the benefits by enhancing the public's KAP about a highly infectious disease such as COVID-19. This awareness also contributes to early healthcare-seeking behavior. Information gathering from experiences of the patients regarding disease occurrence and community preparedness is crucial if further COVID-19 pandemic need to be prevented. In this regard, periodic educational sessions by using the locally adjusted methodology, particularly through the religious scholars and lady healthcare workers, can help to prevent panic among the population, encourage the people to seek early healthcare, and reduce infection and mortality. Based on these results, the healthcare workers and policymakers should consider improving and designing educational programs to help the uneducated women.

Coronavirus disease 2019 is essentially a worldwide catastrophe, so dealing with this pandemic will necessitate collaborative efforts. In Pakistan, the Pakistani government is responsible for effective action in line with the directives of WHO. Overall, other research reports indicate that during this COVID-19 pandemic, it is critical to raise awareness and focus on mental health difficulties of the people. It is advised to the people that they should not be panic and stay calm. Self-efficacy of the individuals and the preventive measures against the COVID-19 can play a crucial role in alleviating mental distress in such outbreaks.

## Limitations

This study, such as other questionnaire-based studies, has a few limitations. First and foremost, we only studied the KAP and mental health in one city in Pakistan, our findings cannot be extrapolated to the entire population of the country. Second, it is impossible to establish a cause-and-effect relationship in such studies.

## Conclusion

In terms of SM use and duration, no gender differences were found. Women had higher levels of psychological issues such as anxiety, stress, and depression compared to men. The male respondents had significantly more knowledge of the COVID-19 compared to female respondents, but the women had significantly better attitudes and practiced COVID-19 prevention measures. Gender is a significant determinant of the psychological distress and KAP concerning the COVID-19. The government has already taken significant steps to limit the spread of the disease; however, much more effort is required to tackle this COVID-19 pandemic.

## Data Availability Statement

The original contributions presented in the study are included in the article/supplementary material, further inquiries can be directed to the corresponding author/s.

## Ethics Statement

The ethical approval was taken from the research and Ethical Review Committee, The University of Faisalabad, Faisalabad, Pakistan (Reference No. TUF/DEAN/2020/56).

## Author Contributions

MR and TA contributed to the conceptualization and design of the study. MR, TA, and MB contributed to the data collection and preparation of the first draft. TA and MB contributed to the software and analysis. MR, TA, XQ, MM, MB, AS, ST, FB, RN, and JH contributed to the editing, reviewing, and proofreading. TA contributed to the guidance and critical discussion. TA and XQ contributed to the supervision of the study. All authors contributed significantly and approved the submitted version for publication.

## Conflict of Interest

The authors declare that the research was conducted in the absence of any commercial or financial relationships that could be construed as a potential conflict of interest.

## Publisher's Note

All claims expressed in this article are solely those of the authors and do not necessarily represent those of their affiliated organizations, or those of the publisher, the editors and the reviewers. Any product that may be evaluated in this article, or claim that may be made by its manufacturer, is not guaranteed or endorsed by the publisher.
